# Coach Rating Combined With Small-Sided Games Provides Further Insight Into Mental Toughness in Sport

**DOI:** 10.3389/fpsyg.2019.01552

**Published:** 2019-07-03

**Authors:** Ben Piggott, Sean Müller, Paola Chivers, Matthew Burgin, Gerard Hoyne

**Affiliations:** ^1^School of Health Sciences, University of Notre Dame, Fremantle, WA, Australia; ^2^Discipline of Exercise Science, Murdoch University, Murdoch, WA, Australia; ^3^Institute for Health Research, University of Notre Dame, Fremantle, WA, Australia; ^4^Western Australian Cricket Association, Perth, WA, Australia

**Keywords:** mental toughness, psychomotor skill, pressure scenarios, perceptual-cognitive-motor skill, sports specific assessment

## Abstract

Literature indicates that mental toughness contributes to successful performance when faced with challenge. This study used an exemplar sport of Australian Rules football to investigate whether skilled performance thrived across increased challenge in small-sided games. Higher (*n* = 14) and lower (*n* = 17) skilled Australian footballers were recruited. First, coaches rated participants’ mental toughness (MTC) using the Mental Toughness Index. Second, participants competed in small-sided games where challenge was manipulated by varying the attacker to defender ratio to create lower and higher pressure scenarios. Decision-making, motor skill execution, and a combined total were measured. MTC rating was higher for higher skilled players. Total score of higher skilled players was significantly superior to lower skilled players in higher and lower pressure scenarios (*p* = 0.003). A “pressure differential score,” calculated to determine whether participants maintained performance across increased challenge, indicated a significant decrease in performance (total score) from lower to higher pressure scenarios for lower skilled (*p* = 0.011), but not for higher skilled (*p* = 0.060) players. Furthermore, MTC scores were predictive of high pressure scenario total scores (*p* = 0.011). Findings suggest higher levels of mental toughness may contribute to maintain performance across the increased challenge of pressure within small-sided games. Practitioners can subjectively rate athlete mental toughness and then structure small-sided games to objectively measure performance under pressure scenarios. This provides an interdisciplinary approach to assess and train psychomotor skill.

## Introduction

In the Australian Football League (AFL) grand final of 2010, both St Kilda and Collingwood had scored 68 points when the siren sounded for full time; a draw. The rules of the competition stated that the final had to be replayed the following week. In the week preceding the rematch, Collingwood coach, Michael Malthouse, penned a newspaper article titled “Mental toughness will decide premiership” (Malthouse, 2010). Collingwood went on to win the grand final replay by 56 points. They were able to perform to a higher level than their opposition, despite the challenge of having already played in a grand final seven days earlier in front of 90,000 spectators, and the adversity of having fatigued and injured players. According to their coach, the success of Collingwood in the replay of 2010 grand final would suggest that mental toughness (MT) played a major role. MT is a concept that indeed warrants further investigation in sports-specific *in situ* settings.

Mental toughness has gained significant interest from the general public, practitioners, and researchers in recent years because it is considered a key factor of superior performance in a variety of domains including sport (Giles et al., 2018). Based upon scientific evidence of MT in a variety of domains such as business, military, education, and sport, the focus has been upon defining the phenomenon and formulation of a theoretical framework from which hypotheses could be tested (Gucciardi et al., 2015). Accordingly, MT has been defined as a personal capacity to produce consistently higher levels of subjective or objective performance, despite everyday challenges, stressors and significant adversities (Gucciardi et al., 2015). Research into MT has also developed and validated several introspective questionnaires in order to measure MT in several domains, and in turn, test and further refine its theoretical framework (Newland et al., 2013). Some of these questionnaires include the Mental Toughness Questionnaire-48 (MTQ48) (Clough et al., 2002), the Sport mental toughness questionnaire (SMTQ) (Sheard et al., 2009), and the Mental toughness index (MTI) (Gucciardi et al., 2015). Therefore, the vast majority of what is known about MT measurement is based upon introspective survey methodology.

Surveys can provide scientists with valuable information about psychological and emotional states of participants, but there has been a call for incorporation of other methodologies that can provide insight to the link between psychomotor skill capability and MT in athletes (Gucciardi et al., 2008, 2013; Gucciardi and Gordon, 2013; Mahoney J. et al., 2014). The broader field of sport psychology has relied predominantly on introspective surveys and interviews, often undertaken sometime after the competition event. This relies on the athlete having to reflect on their own performance, which can be quite subjective. There is scope, however, to utilize an alternative methodology to better understand the link between psychomotor skill performance and MT in sport. For example, Mahoney J. et al. (2014) recommended that a key starting point for future investigation of MT, is objective measurement of performance under manipulation of lower and higher pressure *in situ* contexts in sport. This would enable a quantitative assessment of a component prediction of MT theory through varying degrees of pressure that creates increased challenge to performance. This form of assessment places a participant in a game-like pressure context and would help coaches to identify those players who thrive or succumb to the pressure (Mahoney J. et al., 2014). Accordingly, it has been suggested that a characteristic of mentally tough athletes is for their performance to thrive (i.e., performance maintained to a greater degree) when under increased task pressure or challenge (Jones et al., 2007; Gucciardi et al., 2008).

The importance of MT to superior performance in sport is well recognized among coaches, athletes and sport scientists (Connaughton and Hanton, 2009). Several studies in sport have reported that athletes with higher self-reported scores on MT questionnaires tend to perform better in competition (Drees and Mack, 2012; Newland et al., 2013; Mahoney J.W. et al., 2014; Cowden, 2016). For example, Cowden (2016) reported that amongst professional tennis players, achievement of higher MT scores were correlated with a greater likelihood of winning a national tournament. Drees and Mack (2012) also reported that higher MT scores of high school wrestlers was positively correlated with the season winning percentage. Moreover, Mahoney J.W. et al. (2014) reported a negative relationship such that higher MT scores of adolescent cross-country runners correlated with faster race times. In collegiate basketball players, Newland et al. (2013) reported that MT score partially predicted basketball performance (measured using a metric that combined shot percentage, points, rebounds, assists, steals, turnovers and personal fouls). To our knowledge, there has been no integration of MT survey methodology with systematic manipulation of sports-specific pressure and assessment of perceptual-cognitive-motor skill to further understanding of MT.

Small-sided games (SSG) have been used with several invasion sports such as soccer, field-hockey and Australian rules football (ARF) to assess the perceptual-cognitive-motor skill of athletes (e.g., see Práxedes et al., 2018). Assessment of the correct decision, such as to pass the ball to a teammate or retain possession of the ball, provides a measure of perceptual-cognitive or decision-making skill (Starkes et al., 2004). Alternatively, assessment of the capability to successfully pass the ball to a teammate provides a measure of motor skill execution (Starkes et al., 2004). Recently, Piggott et al. (2019) designed a sports-specific ARF test based upon SSG (six attackers vs. five defenders) in order to assess the perceptual-cognitive-motor skills of Australian footballers. The authors reported that higher skilled players were significantly superior on the total score (decision-making plus execution) compared to lesser skilled Australian footballers. By manipulation of the ratio of defenders to attackers it is possible to increase or decrease pressure in SSG (see Davids et al., 2013), which can then form scenarios to investigate how athletes deal with challenge (as predicted in MT theory) that may affect performance.

This study incorporated existing MT survey methodology with an *in situ* sports-specific test. ARF was used as the exemplar sport skill to further understand the link between MT and psychomotor skill in sport. A sports-specific *in situ* test for ARF was designed based upon SSG that measured participants’ decision-making and motor skill execution under higher and lower pressure scenarios. The purpose of this study was to investigate whether: (i) differences existed between higher and lower skilled ARF players during pressure scenarios of a SSG test, which provided a proxy to test a component of MT theory that performance in terms of perceptual-cognitive-motor skill would thrive (be maintained) under increased pressure, and (ii) whether MTC scores predicted performance scores in the high pressure scenario. Based upon MT theory and the above literature, it was hypothesized that: (a) higher skilled players will perform signficantly better than lower skilled players in both pressure scenarios, (b) higher skilled, rather than lesser skilled players, would better maintain performance across lower to higher pressure scenarios, and (c) MTC questionnaire scores can predicthigh pressure scores inn SSG.

## Materials and Methods

### Participants

A total of 31 male participants were recruited for this study. A higher skilled group (*n* = 14) comprised of one semi-professional West Australian Football League (WAFL) club who participated in the state league. The lower skilled group (*n* = 17) was recruited from two West Australian Amateur Football League (WAAFL) clubs. For a participant to be included in the higher skilled group they had to have played a minimum of one senior WAFL game within the previous two years. The age of the WAFL group was (Mage = 23.0 years, age range: 20–29 years) and the WAAFL group was (Mage = 23.9 years, age range: 19–30 years). Based upon Piggott et al. (2019), a power analysis was conducted using α = 0.05, power (1−β) = 0.8, effect size = 0.15, and two skill groups, which indicated that appropriately 351 trials in total were required. Ethical approval was received from the relevant university committee and participants provided written informed consent.

### Materials and Procedures

#### Mental Toughness

All testing was completed at the end of the pre-season training period and before competition games began for the 2017 season. The first component of this study involved coach rating of each participant using the MTI, an eight item scale that when developed displayed strong factor loadings and composite reliabilities were reported to be excellent (ρ=0.86 *to* 0.89) (Gucciardi et al., 2015). Example items from the scale include *I am able to regulate my focus when performing tasks* and *I am able to execute appropriate skills or knowledge when challenged*. The MTI has been used in previous research with athletes and has been reported to correlate with competition performance (e.g., Gucciardi et al., 2015). The measuring of coach rated MT (MTC) has also been used in previous research (Bell et al., 2013; Cowden et al., 2014) and is a way of overcoming self-attribution bias that can occur when using self-report. To establish face validity for coach rated MT using the MTI, a panel of expert AFL coaches was consulted and all agreed that the questionnaire was an appropriate measure of assessment. A senior member of each of the teams’ coaching staff involved in the study completed the MTC rating for each participant that they coached.

#### Small-Sided Games

The second component of this study involved both skill groups and relates to item 7 on the MTI: *I am able to execute appropriate skills or knowledge when challenged*. This refers in part to MT theory mentioned earlier and was deemed the easiest to manipulate in terms of pressure contexts in a sports-specific *in situ* test. For this study, the previous data set from Piggott et al.’s (2019) was built upon to include another series of SSG involving six attackers versus three defenders. The two series of SSG were termed “higher pressure” (six vs. five) and “lower pressure” (six vs. three), respectively. Both scenarios involved attackers attempting to maintain possession of the ball in a 40 m × 40 m grid using a handpass or kick, whilst defenders tried to spoil or tackle. After a 15 min warm-up, each skill group completed three sets each in attack and defense. The test was umpired by a research assistant with ARF knowledge. Each skill group completed the higher pressure scenario in one session and the lower pressure scenario in another session at least one week apart due to logistics. Each time an attacking player passed the ball by a kick or handball it was recorded as a trial. Decision-making and motor skill execution scores for each disposal (trial), as well as both combined (total score) were coded as objective performance measures. Each session was recorded using a standard 25 Hz video camera (Panasonic SDR-H250, Australia) for later coding (see Piggott et al., 2019, for details of this method).

The scoring system used in both higher and lower pressure scenarios is outlined in Table 1. In relation to item 7 of the MTI, we reasoned that decision-making score reflects “knowledge,” the execution score reflects “skills,” and “challenge” reflects the increase in defenders from three to five in the *in situ* scenarios. The reliability of scoring the scenarios was assessed by inter- and intra-rater reliability. A novice coach scored all trials by viewing the video record of the SSG. Intra-rater reliability was assessed by the same novice coach (two weeks apart) and inter-rater reliability was assessed by an expert ARF coach (Level 3) on 136 (14%) randomly selected trials, across both high and low pressure scenarios, using the video record.

**TABLE 1:** Composite score for test trials.

**Measure**	**Rating**
Decision-making	0 = Incorrect decision
	2 = Next correct decision
	3 = Most correct decision
Execution	0 = Ball does not reach target player at all
	1 = Ball reaches target player but not on full
	2 = Ball reaches target player on full;
	however, the target player had to change
	direction to ensure this occurs
	3 = Ball reaches target player on full
Total	Decision Making + Execution Scores

*Adapted from Piggott et al. (2019). “Small-sided games can discriminate perceptual-cognitive-motor capability and predict disposal efficiency in match performance of skilled Australian footballers.” Journal of Sports Sciences, p. 3.*

#### Data Analysis

Statistical analysis was performed using IBM SPSS version 24 (IBM SPSS Statistics for Windows. IBM Corp., Armonk, NY, United States). Data was checked for normality using the Shapiro Wilk’s test so that the appropriate statistical tests could be employed. Alpha level was set at 0.05.

During the SSG, each time an attacking player passed the ball by a kick or handball it was recorded as a trial, with a total of 980 trials recorded; 423 in the higher pressure scenario and 557 in the lower pressure scenario. The participants’ total score for the SSG was the dependent variable, which equalled the sum of the decision and execution scores for each trial with the maximum score achievable being six. Total score had previously been reported to discriminate superior performance of higher and lower skilled Australian footballers (see Piggott et al., 2019).

Inter- and intra-rater reliability of the scoring of the SSG test was assessed using a two-way mixed intra-class correlation coefficients (ICC) according to Portney and Watkins (2009). The cut-off interpretations used for the ICC values were; less than 0.5, between 0.5 and 0.75, between 0.75 and 0.9, and greater than 0.90 indicative of poor, moderate, good, and excellent reliability, respectively.

Hypothesis one predicted that higher skilled players will perform significantly better than lower skilled players in both pressure scenarios. To address this hypothesis, repeated measures of trials were examined for between skill group differences for total score on the SSG test, in both higher and lower pressure scenarios, using generalised estimating equations (GEE). GEEs use the generalized linear model to estimate more efficient and unbiased regression parameters relative to ordinary least squares regression that accounts for the form of within-subject correlation of responses on dependent variables of many different distributions (Ballinger, 2004). A 2 (skill group) × 2 (pressure scenario) mixed between-within factorial GEE model was used to examine main and interaction effects. Total score was the dependent scale variable in the GEE model. For *post hoc* comparisons, a Bonferroni adjustment was applied. Cohen’s *d* effect sizes were also calculated, with 0.2 considered small, 0.5 medium and 0.8 large effects (Cohen, 1998).

Hypothesis two predicted that higher skilled, rather than lesser skilled players, would better maintain performance across lower to higher pressure scenarios. To address this hypothesis, a mean of the trials’ total score for each participant was calculated for each higher and lower pressure scenarios on the SSG test for both skill groups. A “pressure differential score” was then calculated by subtracting higher pressure scenario mean total score from lower pressure scenario mean total score. For example, if a participant had a lower pressure mean total score of 5.4 and higher pressure mean total score of 4.6, their pressure differential score would be; 5.4 – 4.6 = 0.8. A pressure differential score closer to zero indicated better sustained performance across lower to higher pressure scenarios; which meant participants were able to maintain perceptual-cognitive-motor skills when challenged. A one-sample *t*-test was used to compare the mean pressure differential scores to a no-performance-change score of zero for higher and lower skilled players.

Hypothesis three predicted a relationship between MTC questionnaire scores and SSG high pressure scores. To address this hypothesis, a GEE regression model was used to investigate the relationship between MTC and high pressure scenario total score.

## Results

### Reliability of SSG Test Scoring

Inter-rater reliability for decision (*r* = 0.91, range = 0.88 to 0.94) and execution (*r* = 0.98, range = 0.98 to 0.99) indicated excellent levels of agreement. Intra-rater reliability for decision (*r* = 0.86, range = 0.80 to 0.90) and execution (*r* = 0.96, range = 0.95 to 0.97) also indicated good and excellent levels of reliability, respectively.

### Skill Group Differences for SSG

**FIGURE 1:**
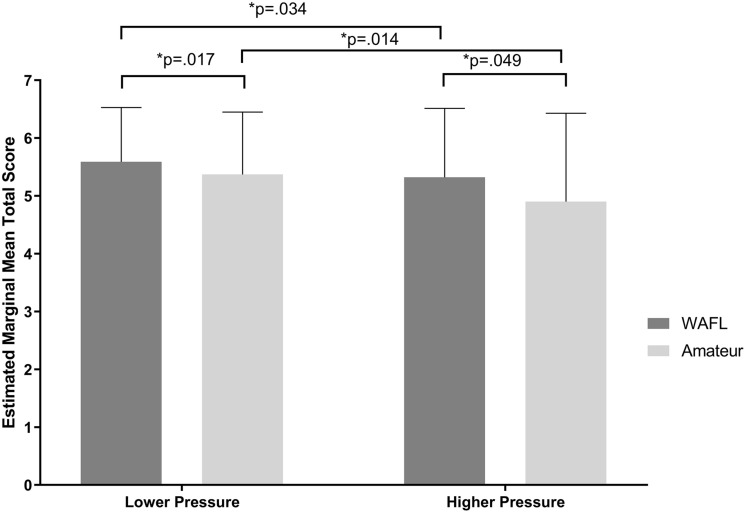
Estimated marginal means for total score in small-sided games test based on GEE model. Pairwise comparisons are depicted ^*^p < 0.05. Error bars represent standard error.

The estimated marginal means derived from the GEE model are presented in Figure 1. The model indicated significantly superior overall performance by higher skilled players over lower skilled players (β = 0.22, SE = 0.07, *p* = 0.003). In addition, performance under the low pressure scenario was significantly higher than under the high pressure scenario (β = −0.47, SE = 0.15, *p* = 0.002). The interaction between skill level and pressure was not significant (β = 0.20, *SE* = 0.18, *p* = 0.282). However, pairwise comparisons revealed a significantly higher total score by higher skilled players (*M* = 5.32, *SE* = 0.08), over lower skilled players (*M* = 4.90, *SE* = 0.14), under the higher pressure scenario (*p* = 0.049, *d* = 0.31). Pairwise comparisons also revealed a significantly higher total score by higher skilled players (*M* = 5.59, *SE* = 0.05), over lower skilled players (*M* = 5.37, *SE* = 0.06), under the lower pressure scenario (*p* = 0.017, *d* = 0.22). When the total score of higher skilled players was compared across lower (*M* = 5.59, *SE* = 0.05) to higher (*M* = 5.32, *SE* = 0.08) pressure scenarios, a significant difference was found (*p* = 0.034, *d* = 0.12). A significant difference was also found for lower skilled players across lower (*M* = 5.37, *SE* = 0.06) to higher (*M* = 4.90, *SE* = 0.14) pressure scenarios (*p* = 0.014, *d* = 0.35).

In relation to performance across scenarios, the pressure differential score indicated that for lower skilled players there was a significant difference compared to a no-performance-change score of zero, *t*(16) = −2.89, *p* = 0.011. This indicates performance decrement for lower skilled players. Conversely, no significant difference was found for pressure differential score for higher skilled players, *t(*13) = −2.06, *p* = 0.060. This indicates maintenance of performance for highly skilled players. There was a medium effect size (*d* = 0.46) for the pressure differential scores between higher (*M* = 0.22, *SD* = 0.40) and lower (*M* = 0.48, *SD* = 0.68) skilled players.

### MTC Scores and Relationship to SSG

The MTC scores for higher and lower skilled groups were (*M* = 43.71, *SD* = 4.41) and (*M* = 42.50, *SD* = 5.37), respectively. There was no significant difference between the two groups for MTC; *t*(26) = 0.519; *d* = 0.24).

When examining the relationship between subjective and objective measures, MTC scores were able to predict high pressure scenario total scores (β = 0.04, SE = 0.01, *p* = 0.011).

## Discussion

The purpose of this study was to integrate methodologies in order to test a component of MT theory that predicts whether an athlete will thrive under pressure situations and maintain performance. This was done in interlinked components; first, a validated questionnaire was used for coaches MT rating of participants. Second, perceptual-cognitive-motor skill differences between higher and lower skilled ARF players were compared across two pressure conditions of a SSG test. Third, the relationships between quantitative and qualitative measures was explored. Fourth, the reliability of scoring the SSG test was checked through inter- and intra-rater reliability. Skill group differences were found in each pressure scenario, with higher skilled players better able to maintain performance across the pressure scenarios. Furthermore, MTC was predictive of performance under high pressure. Therefore, this study made initial steps to address the call for MT research methodology to be extended and incorporate experimental designs that involve systematic manipulation of pressure contexts within an *in situ* setting (Mahoney J. et al., 2014).

Both higher and lower pressure scenarios were able to discriminate skill levels, with higher skilled players performing at a superior level than lower skilled players. Previously, MT research in sport has used only survey methodology to attempt to discriminate between skill levels or categories; such as starters and non-starters in basketball (Newland et al., 2013) and team ranking in tennis (Cowden et al., 2014). This study showed that perceptual-cognitive-motor total score in the lower pressure (six vs. three) scenario was also able to discriminate between higher and lower skilled players. This builds upon Piggott et al.’s (2019) study providing further evidence of the capability of SSG’s to discriminate between closely related skill levels of ARF. The design of tasks that involve differing levels of pressure that are highly representative of the competition environment and can discriminate between skill levels provides an important method to test MT theory. Therefore, supporting evidence was provided for hypothesis one.

A unique aspect of this study, was the calculation of the pressure differential score to test a component prediction of MT theory. The theory predicts that mentally tough athletes are able to thrive under pressure (Jones et al., 2007; Gucciardi et al., 2008). The pressure differential score enabled direct investigation of how participants met this challenge by quantification of the degree to which they maintained performance across increased pressure scenarios. Our study showed that higher skilled players could maintain their perceptual-cognitive-motor skill performance across increased pressure scenarios as evidenced by a lower pressure differential score. In contrast, lower skilled players were less able to maintain their perceptual-cognitive-motor skill performance across increased pressure scenarios as evidenced by an increase pressure differential score. Therefore, supporting evidence was provided for hypothesis two. This addressed the call in the literature for an objective measure of MT under manipulation of lower and higher pressure contexts (Mahoney J. et al., 2014).

The findings of this study have also furthered understanding of a subjective measure of MT and its relationship to an objective measure of perceptual-cognitive-motor skill performance. This is important because Gucciardi and Gordon (2013) stated that there was a lack of evidence supporting the link between MT and an objective measure of performance. The significant predictive relationship between coaches’ MT rating of athletes and *in situ* performance in the higher pressure scenario provides support for the theory that higher levels of MT correspond with higher levels of performance. This evidence supports hypothesis three and is consistent with previous studies that have shown a relationship between self-reported MT and competition performance (Drees and Mack, 2012; Newland et al., 2013; Mahoney J.W. et al., 2014; Cowden, 2016). This indicates that either coach or self-report of MT is relevant to motor skill performance, but our study is unique as we have been able to demonstrate through manipulation of the pressure component in which the task is performed, we showed that MTC has predictive capabilities in relation to *in situ* perceptual-cognitive-motor skill.

## Practical Implications and Limitations

The integration of methods to test theory and evidence presented in this study provides opportunities for practical application in terms of assessment and development of athletes. Accordingly, a multi-method approach to MT could be used (Gucciardi et al., 2013). For example, an ARF coach, in consultation with a sport psychologist, could complete the MTI for an athlete as part of their development. The coach could then discuss their responses with the athlete and how they derived each score per item. On item 7 of the MTI (*I am able execute appropriate skills or knowledge when challenged*), the coach may score the athlete three out of seven and could explain that the athlete needs to improve their decision-making and/or skill execution when under pressure situations in competition. The coach would then work with a sport psychologist to design a six week training plan that develops MT. In addition, the coach assisted by a skill acquisition specialist can design a perceptual-cognitive-motor skill training program for a variety of game-specific pressure contexts. Prior to the commencement of the six week training program, the athlete would complete the SSG games test and may record a high pressure score of 4.0 and low pressure score 5.4 (pressure differential = 1.4). The athlete and the coach could set a goal for the athlete to achieve a pressure differential of <1.0 when retested at the completion of the training program.

A potential limitation of the study is that when using SSG and *in situ* methodology, the number of trials per participant can vary. However, Müller et al. (2015) argue that this variation is representative of the game situation. Previously, we have shown using SSG player involvements (trials) is comparable to player involvement (statistics) in competition (Piggott et al., 2019).

## Conclusion and Future Research

Researchers in the domain of mental toughness have called for an extension of methodology beyond traditional surveys often used in sports psychology research. This study used an *in situ* methodology within ARF to create two scenarios; lower pressure and higher pressure SSG scenarios. These two scenarios were able to discriminate between higher and lower skilled players. Also, the pressure differential score showed that the higher skilled players, but not the lower skilled players, could sustain their performance across lower to higher pressure scenarios. This is in line with a component prediction of MT theory. MTC scores were predictive of total scores in the high pressure scenario, indicating a causal relationship between objective and subjective measures of MT. Therefore, through careful manipulation of a SSG task, a prediction of MT theory was tested, which provides support for further application of SSG in field-based sport settings.

Further research is necessary to replicate these findings with other sports, as well as further probe the causal relationship between mental toughness and perceptual-cognitive-motor skill. In relation to the first point, future research could determine the variance that mental toughness scores account for in the SSG task performance. This will help determine how much mental toughness contributes to performance in SSG. In relation to the second point, a psychological skills training program aimed at improving mental toughness could be compared to a perceptual-cognitive-motor skill training program, with their relative benefits assessed using small-sided game pressure scenarios. A psychological skill intervention has been reported to improve mental toughness and performance in cricket batting (see Bell et al., 2013), but has not specifically been compared to perceptual-cognitive-motor skill training and performance in small-sided game scenarios. Collectively, fruitful opportunities exist to further understand how mental toughness and perceptual-cognitive-motor skills contribute to performance of sports skills.

## Data Availability

The datasets generated for this study will not be made publicly available to maintain confidentiality.

## Ethics Statement

The studies involving human participants were reviewed and approved by Human Research Ethics Committee University of Notre Dame Australia. The patients/participants provided their written informed consent to participate in this study.

## Author Contributions

All authors listed have made a substantial, direct and intellectual contribution to the work, and approved it for publication.

## Conflict of Interest Statement

The authors declare that the research was conducted in the absence of any commercial or financial relationships that could be construed as a potential conflict of interest.

## References

[B1] Ballinger G. (2004). Using generalized estimating equations for longitudinal data analysis. *Organ. Res. Meth.* 7 127–150. 10.1177/1094428104263672

[B2] Bell J. Hardy L. Beattie S. (2013). Enhancing mental toughness and performance under pressure in elite young cricketers: a 2-year longitudinal intervention. *Sport Exerc. Perform. Psychol.* 2 281–297. 10.1037/a0033129

[B3] Clough P. Earle K. Sewell D. (2002). “Mental toughness; the concept and its measurement,” in *Solutions in Sport Psychology*, ed. Cockerill I. (London: Thomson Learning), 32.45.

[B4] Cohen J. (1998). *Statistical Power Analysis for the Behavioral Sciences*, 2nd Edn. Hillsdale, MI: Erlbaum Associates.

[B5] Connaughton D. Hanton S. (2009). “Mental Toughness in Sport: Conceptual and pratical issues,” in *Adances in Applied Sport Psychoology: A Review*, eds Mellalieu S. D. Hanton S. (London: Routledge), 317–346.

[B6] Cowden R. (2016). Competitive performance correlates of mental toughness in tennis: a preliminary analysis. *Perc. Motor Skills.* 123 341–360. 10.1177/0031512516659902 27502244

[B7] Cowden R. Fuller D. Anshel M. (2014). Psychological predictors of mental toughness in elite tennis: an exploratory study in learned resourcefulness and competitive trait anxiety. *Perc. Motor Skills.* 119 661–678. 10.2466/30.PMS.119c27z0 25387038

[B8] Davids K. Araújo D. Correia V. Vilar L. (2013). How small-sided and conditioned games enhance acquisition of movement and decision-making skills. *Ex. Sport Sci. Rev.* 41 154–161. 10.1097/JES.0b013e318292f3ec 23558693

[B9] Drees M. J. Mack M. G. (2012). An examination of mental toughness over the course of a competitive season. *J. Sport Behav.* 35 377–386.

[B10] Giles B. Goods P. Warner D. Quain D. Peeling P. Ducker K. (2018). Mental toughness and behavioural perseverance: a conceptual replication and extension. *J. Sci. Med. Sport.* 21 640–645. 10.1016/j.jsams.2017.10.036 29248306

[B11] Gucciardi D. Gordon S. Dimmock J. (2008). Towards an understanding of mental toughness in Australian football. *J. App. Sport Psych.* 20 261–281. 10.1080/10413200801998556

[B12] Gucciardi D. Hanton S. Gordon S. Mallett C. Temby P. (2015). The concept of mental toughness: tests of dimensionality, nomological network, and traitness. *J. Pers.* 83 26–44. 10.1111/jopy.12079 24428736

[B13] Gucciardi D. F. Gordon S. (2013). “Mental toughness in sport: Past, present, and future,” in *Mental Toughness in Sport: Developments in Theory and Research*, eds Gucciardi D. F. Gordon S. (London: Routledge), 233–251.

[B14] Gucciardi D. F. Mallett C. Hanrahan S. J. Gordon S. (2013). “Measuring mental toughness in sport: Current status and future directions,” in *Mental Toughness in Sport: Developments in theory and research*, eds Gucciardi D. F. Gordon S. (Oxon, NY: Routledge), 108–132.

[B15] Jones G. Hanton S. Connaughton D. (2007). A framework of mental toughness in the world’s best performers. *Sport Psych.* 21:7 10.1123/tsp.21.2.243

[B16] Mahoney J. Ntoumanis N. Mallett C. Gucciardi D. (2014). The motivational antecedents of the development of mental toughness: a self-determination theory perspective. *Int. Rev. Sport Ex. Psych.* 7 184–197. 10.1080/1750984X.2014.925951

[B17] Mahoney J. W. Gucciardi D. F. Ntoumanis N. Mallett C. J. (2014). Mental toughness in sport: motivational antecedents and associations with performance and psychological health. *J. Sport Ex. Psych.* 36 281–292. 10.1123/jsep.2013-0260 24918311

[B18] Malthouse M. (2010). *Mental toughness will decide premiership. The Weekend Australian, 2nd October.*

[B19] Müller S. Brenton J. Rosalie S. M. (2015). Methodological considerations for investigating expert interceptive skill in in situ settings. *Sport, Ex. Perf. Psych.* 4:254 10.1037/spy0000044

[B20] Newland A. Newton M. Finch L. Harbke C. Podlog L. (2013). Moderating variables in the relationship between mental toughness and performance in basketball. *J. Sport Health Sci.* 2 184–192. 10.1016/j.jshs.2012.09.002

[B21] Piggott B. Müller S. Chivers P. Cripps A. Hoyne G. (2019). Small-sided games can discriminate perceptual-cognitive-motor capability and predict disposal efficiency in match performance of skilled Australian footballers. *J. Sports Sci.* 37 1139–1145. 10.1080/02640414.2018.1545522 30424715

[B22] Portney L. G. Watkins M. P. (2009). *Foundations of Clinical Research: Applications to Practice*, 3rd Edn New Jersey, NJ: Pearson Prentice Hall.

[B23] Práxedes A. Moreno A. Gil-Arias A. Claver F. Villar F. D. (2018). The effect of small-sided games with different levels of opposition on the tactical behaviour of young footballers with different levels of sport expertise. *PLoS One* 13:e0190157. 10.1371/journal.pone.0190157 29320519 PMC5761879

[B24] Sheard M. Golby J. van Wersch A. (2009). Progress toward construct validation of the Sports Mental Toughness Questionnaire (SMTQ). *Eur. J. Psych. Assess.* 25 186–193. 10.1027/1015-5759.25.3.186 23968218

[B25] Starkes J. Cullen J. MacMahon C. (2004). “A life-span model of the acquisition and retention of expert perceptual-motor performance,” in *Skill acquisition in sport: Research, theory and practice*, eds Williams M. Hodges N. (London: Routledge), 259–281.

